# White matter hyperintensity in different migraine subtypes

**DOI:** 10.1038/s41598-021-90341-0

**Published:** 2021-05-25

**Authors:** L. A. Dobrynina, A. D. Suslina, M. V. Gubanova, A. V. Belopasova, A. N. Sergeeva, S. Evers, E. V. Gnedovskaya, M. V. Krotenkova

**Affiliations:** 1 3rd Neurological Department, Research Center of Neurology, Volokolamskoe shosse, 80, 125367 Moscow, Russia; 2 Neuroradiology Department, Research Center of Neurology, Volokolamskoe shosse, 80, 125367 Moscow, Russia; 3 Faculty of Medicine, University of Münster, Münster, Germany

**Keywords:** Headache, Migraine

## Abstract

The diagnostic value of white matter hyperintensities (WMH) in different types of migraineare unknown. To evaluate the WMH pattern of different subtypes in migraine patients with no vascular risk factors. 92 migraine patients (73 females, mean age 34.6 ± 8.9; 61 episodic migraine, 31 chronic migraine, 36 migraine with aura, 56 migraine without aura) without vascular risk factors underwent brain MRI (3 T). We also included a matched healthy control group with no migraine (n = 24). The prevalence of WMH in different types of migraine was similar and ranged from 38.7 to 44.4%; the control group showed no WMH at all. Lesions were located within frontal, parietal and temporal lobes (in order of decreasing incidence) in juxtacortical and/or deep white matter. WMH appeared as round or slightly elongated foci with a median size of 2.5 mm [1.5; 3]. Total number, size and prevalence of WMH by lobes and white matter regions were similar between groups, and no interaction with age or sex was found. The number of lesions within the frontal lobe juxtacortical white matter correlated with the age of patients (r = 0.331, p = 0.001) and the duration since migraine onset (r = 0.264, p = 0.012). Patients with different migraine subtypes and without vascular risk factors are characterized by a similar pattern of WMH in the absence of subclinical infarctions or microbleedings. Therefore, WMH have no relevant prognostic value regarding the course of migraine and vascular complications. WMH pattern may be used to differentiate migraine as a primary disorder and other disorders with migraine-like headache and WMH.

## Introduction

Migraine is a chronic neurovascular disorder of the brain with high medical and social significance^1,2^. A migraine diagnosis is based exclusively on clinical features^3^ and until recently, the presence of cerebral lesions on MRI was considered as a criterion to exclude this diagnosis. However, recent epidemiological studies have established that in 29% to 73% of migraine cases, T2 and FLAIR MRI scans reveal white matter hyperintensities (WMH)^4–8^. Most studies demonstrated WMH predominate in migraine with aura^5,6,9,10^ and in chronic migraine^11,12^. The high incidence of WMH became one of the reasons to consider migraine as a risk factor for their development^9,11,13–16^ and highlighted the problem of the diagnostic and prognostic value of WMH in different types of migraine. This question is of high importance, as WMH of vascular origin are a very common MRI sign of small vessels damage due to various reasons, and its severity is associated with cognitive impairment (CI), stroke and disability^17^.

Data regarding the link between WMH and clinical features of migraine, and cerebrovascular complications are contradictory. Several studies demonstrated the association between the WMH burden and the frequency of pain attacks^13,14,18^, duration of disorder^11,19^, its progression over time^6,16,20^, the risk of ischemic stroke^21,22^ and CI^23^, while other studies did not establish any relationship between WMH and severity or duration of migraine^4,24–26^, risk of stroke and other vascular events ^11,24^, CI^9,16,27,28^, and progression of WMH over time^29^. WMH in patients with different types of migraine are predominantly located within the frontal and parietal lobes^9,12,13,18,26,30–32^_,_ in deep and periventricular^6,8,10,13,26^, and sometimes juxtacortical white matter^20,31,32^, with sizes varying from small single to confluent larger lesions^20,30–32^. WMH may be found along with asymptomatic small cerebral infarctions, mainly in the vertebrobasilar system^5,13,18,33,34^ and cerebral microbleedings^35^.

It should be noted that the relationship between WMH and cerebrovascular complications has been established in studies including patients of older age groups^5,9,13,16,36^. Although most of them implemented corrections for cerebrovascular risk factors^5,13,16^, the synergism of migraine and age^30^ in the WMH development cannot be completely ruled out due to neurovascular damage specific for both states^37^. Differentiation of WMH as an MRI sign of migraine in the elderly population is difficult because the lesions are strongly associated with advanced age^38^.The leading cause of WMH, i.e. small vessel disease (SVD) which is associated with age and vascular risk factors, has a prolonged subclinical course^39^. In young patients, establishing WMH as a sign of migraine implies to exclude a whole range of vascular disorders. Thus, the combination of migraine-like headache and WMH may be the earliest manifestation of disorders such as antiphospholipid syndrome, erythrocytosis, CADASIL, MELAS and other hereditary SVD, and small vessel vasculitis^40–42^.

In this study, patients with migraine without vascular risk factors or other causes of cerebrovascular damage were examined to identify the differentiating pattern of WMH in migraine and the potential risk of vascular complications. We assessed the differences in the distribution and severity of WMH, their relationship with clinical features of migraine, the presence of asymptomatic infarctions and microbleedings in episodic or chronic migraine patients with and without aura. Based on the association of MRI signs in migraine with small vessels damage^35^ we used MRI standards for SVD diagnostics^43^ in WMH evaluation. We assessed their localization according to white matter regions considering vascular territories^44^, and size and shape of WMH^45,46^.

The aim of this study was to evaluate WMH pattern and its association with asymptomatic small cerebral infarctions and microbleedings in episodic or chronic migraine patients with and without aura, without vascular risk factors.

## Subjects and methods

**Figure 1:**
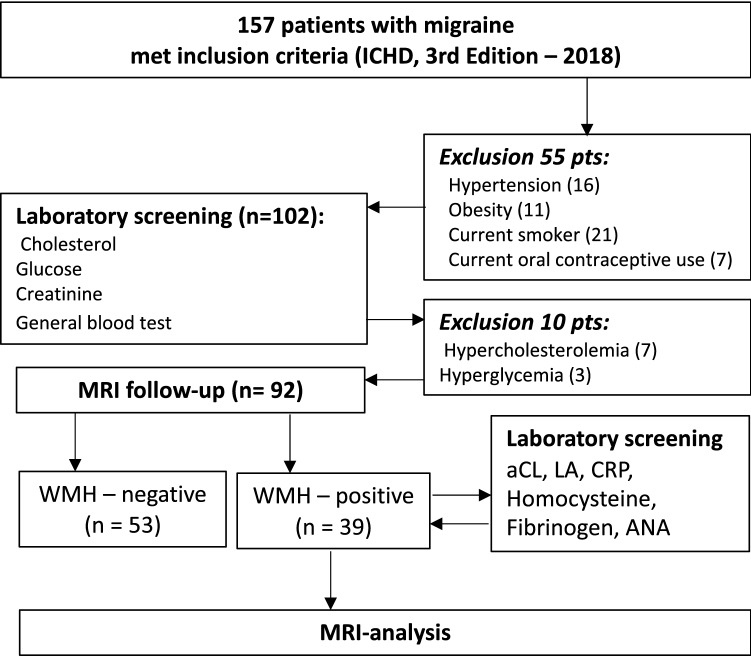
Flow chart of included and excluded subjects. *WMH* White matter hyperintensities, *aCL* Anticardiolipin antibodies, *LA* Lupus anticoagulant, *CRP* C-reactive protein, *ANA* Antinuclear antibodies.

This study used an observational analytic, cross-sectional method and was performed in the Research Center of Neurology. We examined 92 consecutive migraine patients at the outpatient clinic of the Research Center of Neurology, between January 2018 and September 2019. Inclusion criteria were: (1) age 18 to 50 years; (2) headache that meets the criteria for migraine with aura or migraine without aura according to the International Classification of Headache Disorders 3rd edition (2018)^3^. Exclusion criteria were: (1) any other neurological disorder; (2) vascular risk factors such as hypertension (arterial blood pressure ≥ 140/90 mm/Hg), diabetes (glucose ≥ 6.1 mmol/L), obesity (body mass index[BMI] ≥ 30), hypercholesterolemia (cholesterol ≥ 6.2 mmol/L)^47,48^, current smoking, women taking oral contraceptives; (3) other cerebrovascular risk factors, such as thrombophilia, connective tissue disorders, diseases of the blood, heart, kidneys; (4) contraindications for MRI. The flow chart of patients and the reasons for non-inclusion into the study are presented in Fig. 1.

### Clinical data acquisition

We screened 157 patients using a questionnaire form to record their demographic data; progression of the disease including clinical features of migraine (disease duration, attack frequency and duration, attack intensity assessed by visual analogic scale, concomitant symptoms); vascular risk factors (hypertension, diabetes, smoking, hypercholesterolemia, use of oral contraceptives); history of cerebrovascular disorders (transient ischaemic attacks and stroke) and other conditions (connective tissue disorders, thrombophilia, diseases of the blood, heart, kidneys etc.); family history.

Then, the patients underwent clinical examination followed by neurological examination and a general physical examination (obtaining the blood pressure twice and BMI calculation). Thus, migraine diagnosis and its type were established (migraine without aura vs. migraine with aura, episodic migraine (< 15 days with headache) vs. chronic migraine (> 15 days with headache)^3^. Fifty-five patients were excluded due to diagnosis of hypertension (arterial blood pressure ≥ 140/90 mmHg), obesity (BMI ≥ 30), current smokingstatus and women taking oral contraceptives. The remaining 102 patients were evaluated for cholesterol, glucose, and creatinine. Ten patients were excluded due to high glucose and cholesterol levels.

The remaining 92 patients (73 women, 19 men, aged 18 to 50 years, mean age 34.6 ± 8.9 years) underwent brain MRI to identify WMH, and 39 patients with WMH were tested for levels of cardiolipin IgG and IgM antibodies, lupus anticoagulant, CRP, homocysteine, fibrinogen, and antinuclear factor. No laboratory abnormalities were found in any of these patients using standard reference ranges, and all 92 patients were included in the final analysis.

The control group consisted of 24 healthy volunteers of comparable age and gender distribution (20 females, 4 males, mean age 32.8 ± 6.9 years) who were included using the same eligibility criteria, except having a migraine; they also did not have laboratory abnormalities.

### MRI data acquisition

All MRI studies were performed on a 3-T Siemens MAGNETOM Verio scanner (Siemens AG, Erlangen, Germany) using an 8-channel phase-array head coil at the Research Centre of Neurology in Moscow. The imaging protocol included the following anatomical sequences:

1. T2 spin echo (*T2-weighted image*) in the axial view (time repetition (TR)—4000 ms, time echo (TE)—118 ms, slice thickness 5 mm, interslice distance 1.5 mm; duration 2 min 2 s);
2. 3D T1 mpr echo (*T1-weighted image/multiplanar reconstruction*) in the sagittal view to obtain isotropic anatomical data (TR—1900 ms, TE—2.47 ms; slice thickness 1.0 mm; interslice distance 1 mm; duration: 4 min 18 s);
3. 3D FLAIR (*fluid-attenuated inversion recovery*) in the sagittal view with an isotropic voxel (1 × 1 × 1 mm) and a subsequent image reconstruction in 3D (TR—5000 ms, TE—395 ms; duration—6 min 27 s);
4. DWI (*diffusion-weighted image*) in the axial view (TR—6600 ms, TE—100 ms, 25 slices, slice thickness 4 mm, 2 B-factors = 0 and 1000 s/mm^2^, 3 diffusion direction; duration: 2 min 07 s);
5. SWI (*susceptibility-weighted imaging*) in the axial view (TR—28 ms, TE—20 ms, 72 slices, slice thickness 1.2 mm, study time: 6 min 38 s).

WMH, subclinical infarctions, lacunar infarctions and microbleeds were evaluated using STRIVE criteria^43^. Images were visually assessed separately by two neuroradiologists with 14 years of experience as a neuroradiologists, blinded for scan-sequence and for patients’ clinical characteristics (diagnosis). In case of disagreement about absence or presence of lesions, decision was made by consensus.

WMH were assessed separately in the frontal, parietal, temporal and occipital lobes, in the subcortical structures and brainstem, and in the cerebral hemispheres—in the juxtacortical white matter (up to 4 mm from the edge of the cortex and white matter boundary), periventricular white matter (up to 13 mm from the walls of the lateral ventricles) and the deep white matter (area between the periventricular and the juxtacortical white matter)^43^. WMH severity was assessed according to the size (Me [Q25%; Q75%]) (mm) and number of lesions (no focal WMH, ≤ 3, 4–7, ≥ 8).

There were no any subclinical infarctions or microbleedings in the study group, so their assessment criteria are not provided.

The study was conducted in accordance with the Helsinki Declaration and approved by the Local Ethics Committee of the Research Center of Neurology. All participants provided written informed consent after the experimental procedure had been fully explained.

### Data analysis

Descriptive statistics for categorical variables and ordinal variables included number and percentage; for continuous variables arithmetic mean, standard deviation, median, and quartiles were used. All comparisons were performed using two-sided t-tests. The null hypothesis was rejected at p-level of 0.05. Odds ratio and 95% confidence intervals were also calculated. Qualitative variables were estimated using Pearson’s chi-squared test and Fisher’s exact test. Depending on the data distribution, quantitative variables were estimated using t-test or Mann–Whitney test, or one-way ANOVA or Kruskal–Wallis test for more than two independent samples. The relationship between variables was assessed using Spearman's rank correlation. In order to estimate an impact of clinical characteristics of migraine, i.e., aura and frequency of migraine episodes, on the number of WMH, a General Linear Model (GLM) was implemented, with sex and age of the patients used as independent variables. Statistical analyses were performed with IBM SPSS 23.0 software.

## Results

The general characteristics of the study groups, i.e. migraine with aura (n = 36), migraine without aura (n = 56), episodic migraine (n = 61), chronic migraine (n = 31) and control group (n = 24) are shown in Table 1. Since the control group did not show any MRI abnormalities at all, we only analyzed the risk factors and the clinical and demographic data.

**Table 1:** Characteristics of migraine patients and subjects of the control group; there were no significant differences.

	Migraine with aura (n = 36)	Migraine without aura (n = 56)	Episodic migraine (n = 61)	Chronic migraine (n = 31)	Control (n = 24)
Female sex (n, %)	28 (78%)	45 (80%)	48 (79%)	25 (81%)	20 (83%)
Age, years (mean ± SD)	33.2 (8.5)	35.5 (9.1)	33.5 (8.6)	36.5 (9.3)	32.8 (6.9)
Systolic blood pressure, mm Hg (mean ± SD)	107.5 (11.4)	106.7 (11.8)	105.4 (10.7)	110.2 (12.7)	106.5 (13.5)
Diastolic blood pressure, mm Hg (mean ± SD)	70.8 (8.5)	69.2 (8.8)	68.3 (8.1)	72.9 (9.1)	70.8 (8.3)
Body mass index, kg/m^2^ (mean ± SD)	25.79 (2.64)	25.51 (2.66)	25.43 (2.68)	25.99 (2.58)	23 (5.6)
Glucose level, mmol/L (mean ± SD)	5.0 (0.6)	5.0 (0.5)	4.9 (0.6)	5.1 (0.5)	5.2 (0.8)
Cholesterol level, mmol/L (mean ± SD)	5.3 (0.6)	5.2 (0.7)	5.2 (0.7)	5.2 (0.7)	4.5 (1.2)
Family history of migraine (n, %)	25 (69%)	32 (57%)	41 (67%)	16(52%)	–
Disease duration (years) (Me [Q25%; Q75%])	13 [8.5; 21.5]	15.5 [10.5; 22.5]	14 [6; 21]	16 [11; 24]	–
**Treatment**					
Acute (during pain attacks)					
Triptanes	17 (47%)	29 (52%)	32 (52%)	14 (45%)	–
Nonsteroidal anti- Inflammatory drugs	14 (39%)	28(50%)	26 (43%)	16 (52%)	–
Other	12 (33%)	26 (46%)	23 (38%)	15 (48%)	–
Prophylactic					
Antidepressants	13 (36%)	24 (43%)	22 (36%)	15 (48%)	–
β-Blockers	0	3 (5%)	2 (3%)	1 (3%)	–
Anticonvulsants	0	6 (11%)	2 (3%)	4 (13%)	–
**MRI findings**					
WMH (n, %)	16(44.4%)	23 (41.0%)	27 (44.2%)	12 (38.7%)	–
Silent infarct-like lesions	–	–	–	–	–
Microbleeds	–	–	–	–	–

The comparison of groups with migraine and control subjects demonstrated no significant differences by sex, age, body mass index, glycemia and total cholesterol levels; within the migraine groups there were also no differences between the types of migraine by family history, duration of the disease and treatment approaches.

The MRI findings included only WMH. There were no subclinical ischemic changes or microbleedings. WMH were observed in 41.0% of migraine without aura patients and in 44.4% of migraine with aura patients (p = 0.991, OR = 0.952; 95% CI 0.408–2.224), and in 44.2% of episodic migraine patients and in 38.7% of chronic migraine patients (p = 0.379, OR = 0.455; 95% CI 0.191–1.138).

**Figure 2:**
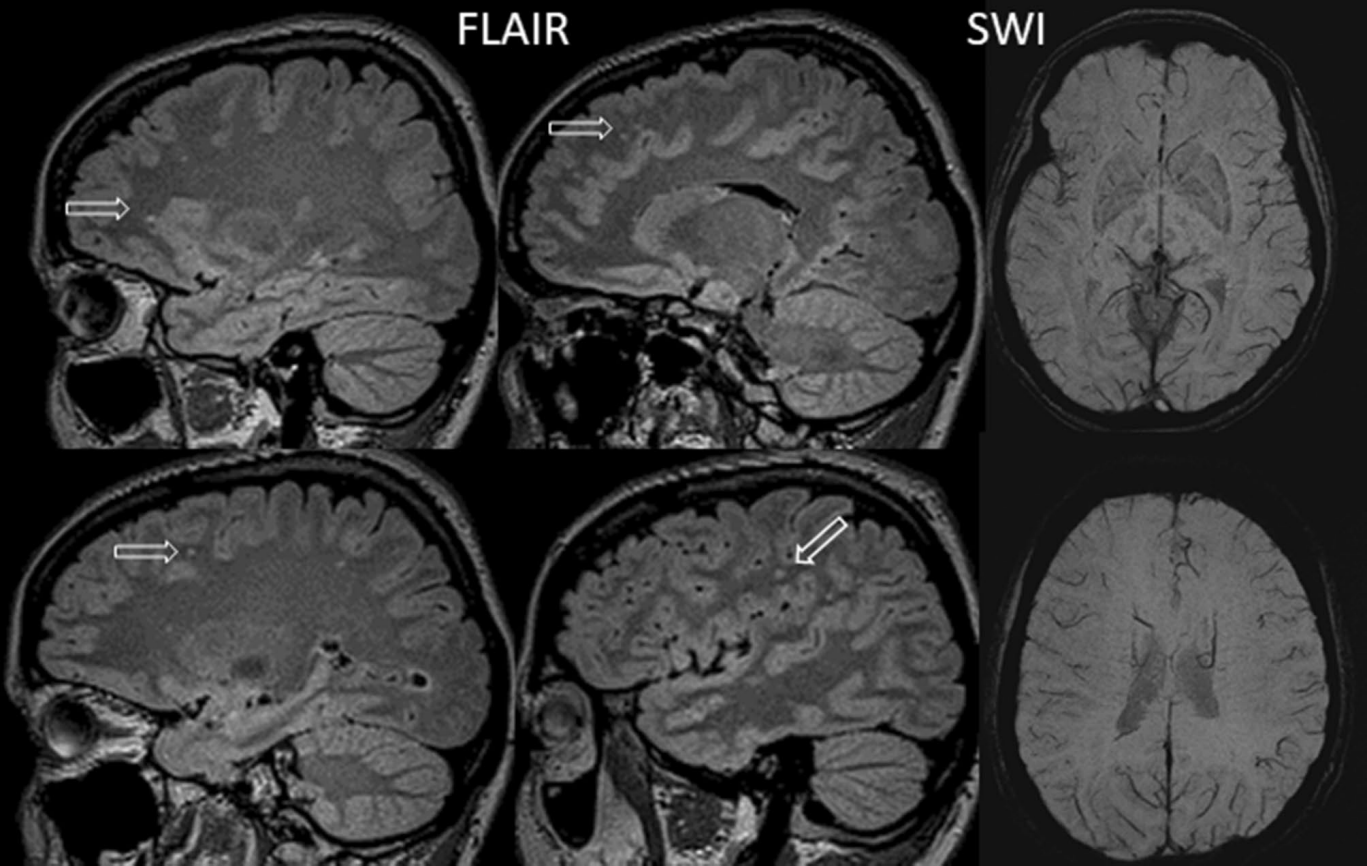
33-year-old female patient, presented with migraine with aura for 10-year duration. Sagittal FLAIR MRI image shows punctate hyperintensities in the white matter within the frontal and parietal lobes (1–2 mm). There are no microbleeds on SWI images.

In all types of migraine, WMH were found only in frontal, parietal, and temporal lobes. In all cases the lesions were focal, with round or slightly elongated shape, median size of 2.5 mm [1.5; 3]; the location was primarily juxtacortical and/or within the deep white matter (Fig. 2), except for two patients with episodic migraine who had single periventricular WMH.

**Figure 3:**
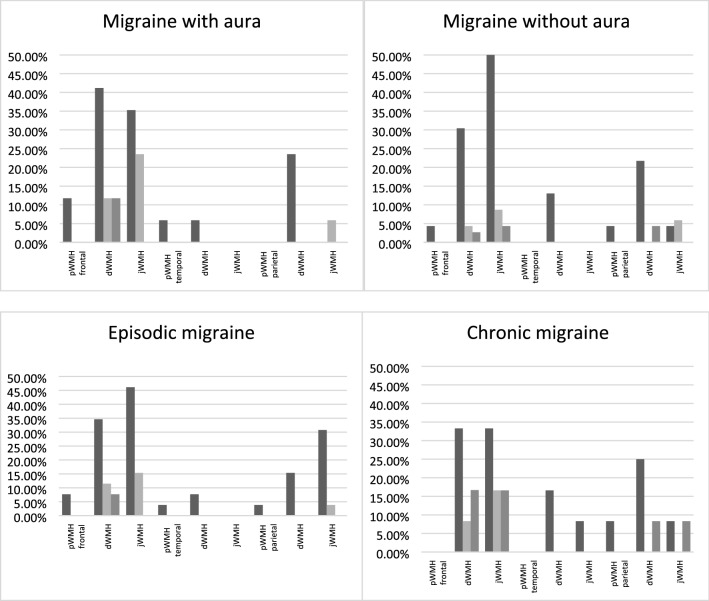
Characteristics of white matter hyperintensities in patients with different types of migraine. There were no significant differences. number of WMH: Dark gray < 3, light gray 4–7, gray  > 8. *pWMH* periventricular white matter hyperintensities, *dWMH* deep white matter hyperintensities, *jWMH* juxtacortical white matter hyperintensities.

The distribution of WMH location by cerebral lobes in migraine patients is shown in Fig. 3. The majority of WMH were found within the frontal lobe: 86.9% (20 out of 23 patients with WMH) of migraine without aura patients; 93.7% (15 out of 16) of migraine with aura patients; 44.4% (12 out of 27) of episodic migraine patients; 100% (12 out of 12) of chronic migraine patients. WMH within the parietal lobe were found in: 30.4% (7 out of 23 patients with WMH) of migraine without aura patients; 43.7% (7 out of 16) of migraine with aura patients; 40.7% (11 out of 27) episodic migraine patients; 25% (3 out of 12) chronic migraine patients. WMH within the temporal lobe were observed in: 13% (3 out of 23 WMH) of migraine without aurapatients; 18.7% (3 out of 16) of migraine with aura patients; 11.1% (3 out of 27) episodic migraine patients; 25% (3 out of 12) chronic migraine patients.

There were no significant differences in the severity of WMH in various cerebral lobes and in the size of WMH between migraine patients with versus without aura, and in patients with episodic versus chronic migraine. The General Linear Model approach did not suggest any differences in the total number of WMH between groups, and no interaction between the presence and absence of aura, and between episodic and chronic course with age and sex of patients.

Assessment of association between the number of WMH within various cerebral lobes and clinical manifestations of migraine demonstrated correlations only between the number of juxtacortical WMH within the frontal lobe and age of the patient (r = 0.331, p = 0.001) and age of migraine onset (r = 0.264, p = 0.012).

## Discussion

First, we could replicate the finding that patients with migraine show a higher incidence of WMH than age- and sex-matched healthy control subjects. This finding is however unspecific since both migraine patients and healthy subjects did not show any infarctions or bleedings.

The study was primarily aimed to clarify the role of WMH in differentiating migraine subtypes and its relation to clinical characteristics of migraine and MRI signs of cerebrovascular lesions, i.e. asymptomatic infarctions in young patients without vascular risk factors and other causes of cerebrovascular diseases. Eligibility criteria were designed to eliminate patients with any possible causes of WMH, subclinical small brain infarctions and microbleedings except migraine. The rationale for the study was the ambiguity of evidence supporting diagnostic and prognostic significance of WMH in migraine^11,13,15^ that may play a role in differential diagnosis and risk assessment of vascular complications. WMH of vascular origin are the most widespread and non-specific MRI sign^17,49–52^. It is difficult to differentiate WMH as a sign of migraine or as of vascular origin, and this may partially explain the inconsistency between the results of previous studies.

In this study, the incidence of WMH in different migraine subtypes was 38.7% to 44.4% that complies with the middle position within the previously reported values between 29 and 73%^4–8^. The incidence of WMH was non-significantly higher in migraine with aura patients (44.4%), similarly to the results of other studies^5,6,9,10^. No significant differences in the incidence of WMH, its distribution across cerebral lobes and size of lesion between groups were found. Universal differentiating features for all types of migraine were: predominance of WMH within the frontal lobe and a lower incidence of lesions within the parietal and temporal lobes and their absence within other lobes; equal incidence of juxtacortical and deep location along with less frequent periventricular localization; small (2.5 [1.5; 3] mm) lesion size; the absence of a tendency to merge. Previous studies have reported not only the localization of WMH within frontal, parietal and temporal lobes^9,12,13,18,26,30–32^, but also their infratentorial and occipital location^11,18,30,34^, the involvement of periventricular white matter^6,8,10,13,26^, various sizes of WMH, including confluent lesions^31^.

It should be noted that our approach to evaluate the white matter allowed us to conclude that WMH in migraine are characterized by juxtacortical localization which was previously mentioned only in few studies^20,31,32^. We also demonstrated a direct association of the increase of juxtacortical WMH lesions within the frontal lobe with age of the patients and duration of the disorder which most probably indicates a role of mechanisms which determine the frontal localization of pain during migraine attack, and the extravasation liquid part of the blood during an attack, in the development of these lesions.

Thus, the pattern of WMH in our population of young migraine patients without vascular risk factors and other causes of cerebrovascular lesions differs from the results of other studies that did not implement such restrictions in patient selection. These differences include the absence of the following signs of WMH: disseminated distribution; periventricular localization; absence of tendency to confluence (i.e., MRI signs described in older migraine patients and signs of age-dependent SVD)^43,53^. The influence of age and vascular factors on the disseminated distribution of WMH in migraine was also confirmed in a recent study by Meilan et al. Among the investigated factors, only the age > 45 years was associated with the severity of WMH, while the type of migraine and the frequency of attacks did not^26^.

Despite different pathophysiological mechanisms underlying different migraine subtypes^54^, they are characterized by similar pattern of WMH. This may be explained by a presence of a common link of WMH pathogenesis, maybe associated with vasodilation and plasma extravasation. In our study, we did not find any relationship between severity of WMH and the severity of migraine as it was demonstrated in previous studies^4,11,13,14,18,19,24–26^. The exclusion of vascular risk factors allowed us to suggest that there were no other causes leading to the increased alterations of the blood–brain barrier other than migraine; therefore, it can be assumed that the underlying mechanisms of WMH pathogenesis include individual properties of the endothelium associated with high vascular wall alteration. This predisposition may be related to a disturbance in the production of the Vascular Endothelial Growth Factor (VEGF). It affects the vascular wall and leads to an increase in the level of nitrogen oxide by stimulating the nitrogen oxide synthase with consequent vasodilatation^55^. Earlier, elevated VEGF levels during a migraine attack^56^ and decreased VEGF levels during the interictal period^57^ were demonstrated, as well as the association between the genetic profile of VEGF and different sensitivity to migraine^58^. The revealed similarity of WMH pattern in different migraine types confirms that specific features of activation and sensitization of trigeminovascular pathways, brain stem and diencephalic nuclei play an important role in the development of various migraine subtypes^59,60^.

In our study groups with different types of migraine, no subclinical brain infarctions were seen, this is consistent with the role of vascular risk factors in their development. This may be also somewhat supported by the fact that in a Cerebral Abnormalities in Migraine, an Epidemiological Risk Analysis study (CAMERA), which established an association between migraine and asymptomatic infarctions in the posterior circulation, the age of the patients was higher^5,13,18,33,34^. We also did not detect any microbleedings, previously described in older patients with migraine^35^. Although in the aforementioned study microbleedings were more common in migraine patients than in the control group^35^, it should be noted that the proportion of patients with hypertension, a leading cause of small vessel damage, was also significantly higher migraine patients. Therefore, it could not be confirmed that migraine was the sole cause for the higher incidence of microbleedings in this group. Since the leading mechanism of microbleedings is the increased alterations of blood–brain barrier, which is typical for both the headache phase of migraine attacks^54^ and SVD (including hypertensive and other age-dependent types of this disorders)^50–53^, the combined influence of these conditions cannot be excluded either.

Our study had some limitations. The cross-sectional nature of the study does not allow making a conclusion about at what stage the formation of WMH occurred and what conditions contributed to this, as well as what effect has the preventive treatment on the increase in WMH.

## Conclusion

Thus, young patients with both episodic and chronic migraine with and without aura and without vascular risk factors have a similar pattern of WMH: rare small-sized (2.5 [1.5; 3] mm) foci located within the frontal, parietal and temporal lobes (in order of decreasing incidence) in juxtacortical and/or deep white matter. These features may be used to differentiate migraine as primary disorder and others disorders with migraine and migraine-like headache and WMH. The lack of association between WMH in patients without vascular risk factors with the subtype of migraine, with frequency and severity of attacks, with duration of the disorder, and with subclinical brain infarctions and microbleedings indicates a very low to no prognostic value of WMH considering the course of migraine and development of vascular complications.

## Data Availability

Raw data were generated at Research Center of Neurology. The data that support the findings of this study are available from the corresponding author upon reasonable request. Clinical, neurovisualization and statistical data will be available upon request from any qualified investigator.
